# Plasma Proteome Responses in Salmonid Fish Following Immunization

**DOI:** 10.3389/fimmu.2020.581070

**Published:** 2020-10-08

**Authors:** Fiona K. Bakke, Milena M. Monte, David A. Stead, Dwight R. Causey, Alex Douglas, Daniel J. Macqueen, Helen Dooley

**Affiliations:** ^1^School of Biological Sciences, University of Aberdeen, Aberdeen, United Kingdom; ^2^Aberdeen Proteomics, The Rowett Institute, University of Aberdeen, Aberdeen, United Kingdom; ^3^The Roslin Institute and Royal (Dick) School of Veterinary Studies, University of Edinburgh, Edinburgh, United Kingdom; ^4^Department of Microbiology and Immunology, Institute of Marine and Environmental Technology (IMET), University of Maryland School of Medicine, Baltimore, MD, United States

**Keywords:** plasma, salmonid, immunity, proteome, liquid chromatography-mass spectrometry, trout, immunoglobulin M

## Abstract

Vaccination plays a critical role in the protection of humans and other animals from infectious diseases. However, the same vaccine often confers different protection levels among individuals due to variation in genetics and/or immunological histories. While this represents a well-recognized issue in humans, it has received little attention in fish. Here we address this knowledge gap in a proteomic study of rainbow trout (*Oncorhynchus mykiss*, Walbaum), using non-lethal repeated blood sampling to establish the plasma protein response of individual fish following immunization. Six trout were immunized with adjuvanted hen egg-white lysozyme (HEL) and peripheral blood sampled at ten time points from day 0 to day 84 post-injection. We confirm that an antigen-specific antibody response to HEL was raised, showing differences in timing and magnitude among individuals. Using label-free liquid chromatography-mass spectrometry, we quantified the abundance of 278 plasma proteins across the timecourse. As part of the analysis, we show that this approach can distinguish many (but not all) duplicated plasma proteins encoded by paralogous genes retained from the salmonid-specific whole genome duplication event. Global variation in the plasma proteome was predominantly explained by individual differences among fish. However, sampling day explained a major component of variation in abundance for a statistically defined subset of 41 proteins, representing 15% of those detected. These proteins clustered into five groups showing distinct temporal responses to HEL immunization at the population level, and include classical immune (e.g. complement system members) and acute phase molecules (e.g. apolipoproteins, haptoglobins), several enzymes and other proteins supporting the immune response, in addition to evolutionarily conserved molecules that are as yet uncharacterized. Overall, this study improves our understanding of the fish plasma proteome, provides valuable marker proteins for different phases of the immune response, and has implications for vaccine development and the design of immune challenge experiments.

## Introduction

Vaccination plays a critical role in protecting humans and other animals from infectious diseases (e.g. (1–4)). While a perfect vaccine will impart full protection to every member of a targeted population, in reality this is limited by the inherent variability among individuals, including genetics and distinct environmental and/or immunological histories. Indeed, it is well recognized that humans show high variability in immune phenotypic parameters (e.g. serum protein levels) and responses (e.g. cytokine induction), which can largely be explained by non-heritable environmental influences (5, 6). However, the extent of individual-level variation in the immune response remains poorly defined in most species. This includes fishes used in aquaculture, an industry that plays a crucial role in global food provision and economic security, yet remains threatened by infectious disease outbreaks (e.g. (7)).

Vaccination is applied broadly in aquaculture (4), and there remains a need to develop new and improved vaccines for many species and diseases, along with less labor-intensive modes of administration (3). An important part of the development pipeline is vaccine efficacy testing, which can be assessed directly *via* the extent of protection (e.g. using disease challenge tests), or indirectly, by studying changes in immune parameters, for example by confirming an antigen-specific antibody response (8, 9) or recording changes in the expression of immune molecules (e.g. (10, 11)). Such work usually relies on the terminal sampling of tissues from large numbers of fish, at different time points, to adequately capture the response dynamic. However, underlying such studies is the assumption that individuals in a population will show similar immune responses (12). A step towards testing this assumption in salmonid species was the development of methods for repeated non-lethal sampling of blood from the same fish (12, 13). This approach can help elucidate immunological variation between individuals, either in their constitutive state or following immunization/disease challenge.

The current study tests the hypothesis that rainbow trout, a commercially important salmonid fish, shows a high degree of individual variation in response to immunization. By combining non-lethal blood sampling with high-throughput proteomics, we sought to document global changes in plasma protein abundance in individual fish following a common immunization protocol. The plasma proteome provides a strong index of biological status (14), including in fish (e.g. (15, 16)), and can be analysed using liquid chromatography-mass spectrometry (LC-MS) using small blood samples (e.g. (17)). Recent work has applied such approaches in salmonids (18), including to document changes in plasma protein abundance during sea water adaptation in rainbow trout (19). Such approaches require a comprehensive protein database for the target species and enable simultaneous identification and quantification of hundreds to thousands of proteins per experiment (20–23).

Our study design involved the immunization of a small cohort of rainbow trout with adjuvanted hen egg-white lysozyme (HEL) followed by sequential blood sampling of every individual at ten points across a 12-week timecourse. After verifying an antigen-specific antibody response was generated by each individual, high-throughput LC-MS proteomics was used to monitor changes in plasma protein abundance in the same animals. Our analyses revealed the extent of individual versus population-wide responses to the same immunization protocol, as well as providing a detailed characterization of a salmonid plasma proteome.

## Materials and Methods

### Immunization Protocol and Plasma Sampling

All procedures were conducted following the UK Home Office ‘Animals and Scientific Procedures Act 1986; Amendment Regulations 2012’ on animal care and use, with prior ethical approval from the University of Aberdeen’s Animal Welfare and Ethical Review Body (AWERB). Rainbow trout (245.6 ± 0.64 g; 27.6 ± 0.19 cm; mixed sex) were purchased from Almondbank Hatchery, Perthshire. The size of fish was chosen to ensure we could collect enough plasma per animal to perform technical optimization and data acquisition without exceeding 10% blood volume taken from any individual per month (13). The fish were maintained in 1-metre diameter tanks containing continuously circulating fresh water at 14±1°C in the aquarium facility of the School of Biological Sciences, University of Aberdeen, and were fed twice daily with standard commercial pellets (EWOS, Scotland). Fish were sedated with 2-phenoxyethanol (Sigma Aldrich) prior to any experimental procedure and daily monitoring undertaken for the duration of the experiment. Following PIT tagging fish were allowed 1 week to recover prior to immunization. Six fish were immunized intraperitoneally (IP) with hen egg-white lysozyme (HEL) emulsified in an equal volume of Complete Freund’s Adjuvant (CFA), and one control fish was immunized with CFA only. HEL is a model antigen that has been used for immunization studies in several other vertebrate species (e.g. (24–26)). We can be certain that our study animals have no prior exposure to this antigen and know from other species the response to HEL is T-dependent and therefore a good proxy for the response we would wish to raise when developing an effective vaccine. Blood samples were drawn on days 0, 7, 14, 21, 28, 35, 42, 56, 70, and 84 post-immunization into syringes containing 100μl porcine heparin reconstituted to 1,000 U/ml in PBS. Plasma was aliquoted into low protein binding tubes, flash frozen, and stored at -80°C prior to analysis.

### Measurement of Antigen-Specific IgM

**Figure 1:**
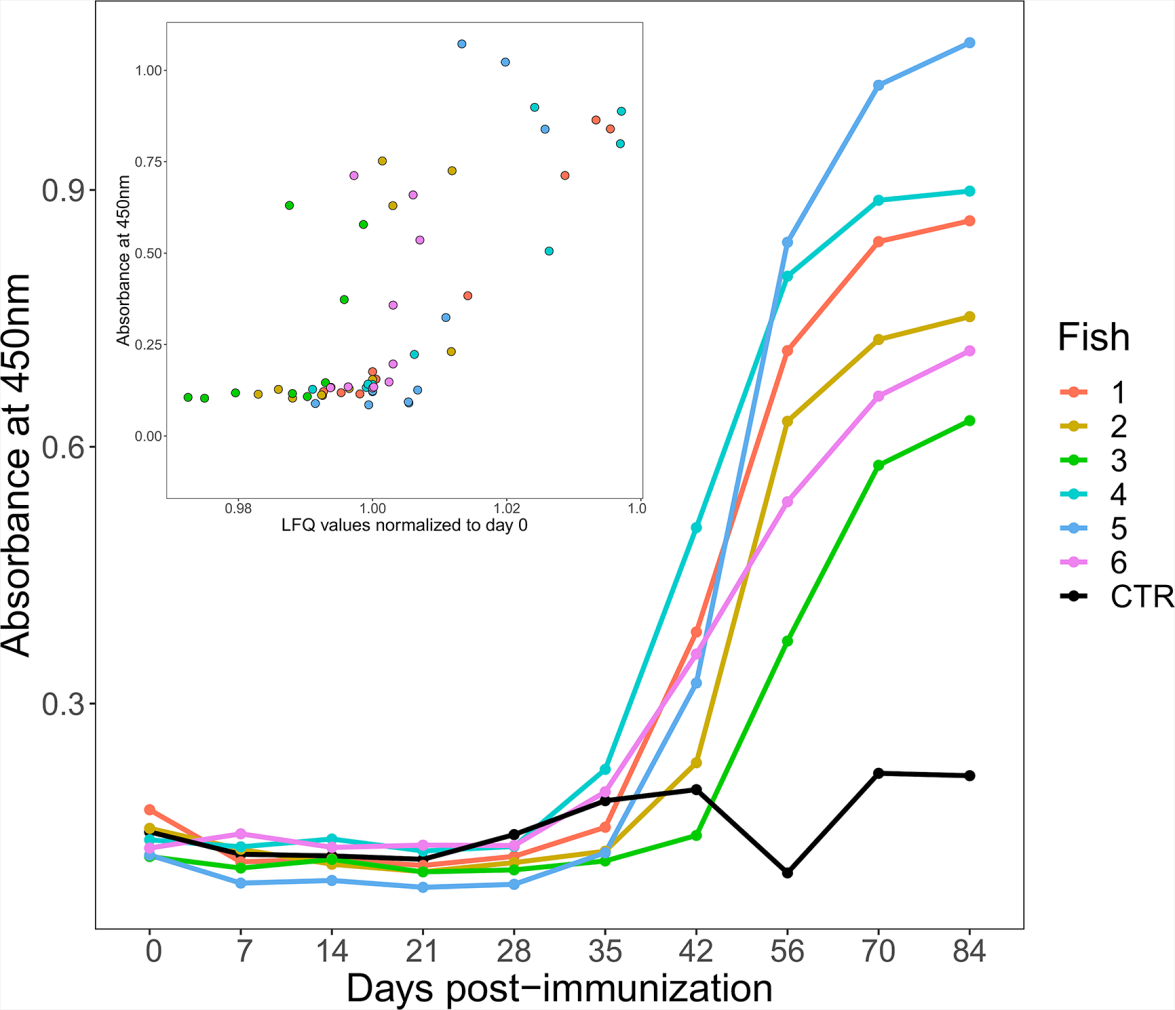
Changes in antigen-specific IgM levels following immunization of six rainbow trout (coloured lines) to HEL-CFA as compared to the CFA-only control fish (black line). The data shown are representative of duplicate technical replicates. The insert shows a positive correlation between antigen-specific and total IgM levels estimated by ELISA and label free proteomics, respectively (Spearman’s Rho = 0.71, P <0.001).

ELISA plates were coated for 48 h at 4°C with 100 μl/well of HEL diluted in 0.05 M carbonate-bicarbonate (pH 9.6) buffer to 10 μg/ml. Wells were emptied, washed once with 200 μl PBS containing 0.05% Tween20 (PBST), and blocked with 200 μl 3% fat-free milk solution (MPBS) for 3 h at room temperature. Plasma samples were pre-diluted 1/10 in PBS then three-fold dilutions performed so that titration curves could be obtained for each sample. Wells were washed with PBST x3, plasma samples added at 100 μl/well then incubated overnight at 4°C. Wells were washed three times with PBST then 4C10 anti-salmonid IgM monoclonal supernatant (27), diluted 1:1 in PBS, was added at 100 μl/well and the plates incubated overnight at 4°C. After three washes with PBST, anti-mouse HRP-conjugated antibody (Sigma Aldrich), diluted 1:1000 in MPBS, was added at 100 μl/well and incubated for 2 h at room temperature. Following three further washes with PBST, binding was detected by the addition of 100 μl/well TMB solution; the reaction was stopped by the addition of 50 μl/well 1M H_2_SO_4_, and the plate read at 450 nm. Data were normalized across the plates, and titration curves plotted for each timepoint for each animal. To illustrate the data more easily we picked a dilution where signal was subsaturated for all samples, in this case the 1/270 dilution, and used this to plot absorbance at 450nm against time (as shown in **Figure 1**). Plasma from our CFA-only immunized animal was used as the negative control. The following technical controls were also performed; plasma samples on an irrelevant target (i.e. ELISA wells both coated and blocked with MPBS), PBS in place of plasma, and monoclonal antibodies raised against irrelevant targets in place of the 4C10 monoclonal.

### LC-MS Analysis

Plasma samples were prepared for proteomic analysis at Aberdeen Proteomics (University of Aberdeen) for the same n=6 trout individuals assayed by ELISA (as above). 1 μl of plasma was diluted with 99 µl 50mM ammonium bicarbonate and proteins were reduced in 2mM dithiothreitol for 25 min at 60°C, S-alkylated in 4mM iodoacetamide for 30 min at 25°C in the dark, digested with porcine trypsin (sequencing grade, Promega) overnight at 37°C, then freeze-dried. The protein pellets were dissolved in 40 µl 0.1% TFA and desalted using ZipTip µ-C18 stage tips (Merck Millipore) following the manufacturer’s instructions. The eluted peptide solutions were dried and dissolved in 10 µl LC-MS loading solvent (98 parts UHQ water: 2 parts acetonitrile: 0.1 parts formic acid). Samples were transferred to a 96-well microtitre plate ready for injection into an UltiMate 3000 RSLCnano LC system (Thermo Scientific Dionex) coupled to a Q-Exactive Hybrid Quadrupole Orbitrap MS system (Thermo Scientific). The LC was configured for pre-concentration onto a PepMap RSLC C18 50 µm x 25 cm column (Thermo Scientific P/N ES802) fitted to an EASY-Spray ion source (Thermo Scientific). The loading pump solvent was UHQ water: acetonitrile: formic acid (98: 2: 0.1) at a flow rate of 10 µl/min; nano pump solvent A was UHQ water: formic acid (100: 0.1); nano pump solvent B was acetonitrile: UHQ water: formic acid (80: 20: 0.1). The LC gradient was programmed to increase the proportion of solvent B from 3% to 10% between 5 and 15 min, from 10% to 40% between 15 and 95 min, from 40% to 80% from 95 to 100 min and hold for 10 min before re-equilibration of the nano-column in 3% solvent B for 25 min. MS data acquisition was started at 10 min into the LC gradient, 5 min after switching the flow through the pre-column, and continued for a total of 100 min.

### Computational Proteomics

MS data were uploaded to MaxQuant v1.5.3.30 (28) together with a reference database containing all protein sequences (n=71,625) predicted in the current rainbow trout RefSeq genome assembly (NCBI accession: GCA_002163495.1) (29). Despite representing a comprehensive annotation of proteins, we noted a poor representation of immunoglobulin (Ig) M (IgM) proteins. As we were interested in quantifying total IgM in addition to antigen-specific IgM, we supplemented the reference database with 317 *O. mykiss* IgM proteins downloaded from the NCBI nr protein database. The Andromeda peptide search engine within MaxQuant (21) was used to match the mass spectra of all detected peptides against the reference protein database, using the label-free quantification method. Digestion type was set to “trypsin” and two missed cleavages were permitted to maximize peptide detection if tryptic digestion was incomplete. Variable modifications of methionine oxidation and N-terminal acetylation were allowed. To increase quantitative accuracy, the Fast LFQ option was not selected. “Match between runs” was used to maximise peptide detection; any features with retention times within 0.7 min and mass within the mass tolerance of peptides identified during previous runs (30) were assigned the same peptide identity. The false discovery rate was set at 0.01, using a target-decoy based search applied at both peptide and protein group levels, which calculated the probability that identified peptides and proteins were false hits (31).

The Andromeda platform within MaxQuant generated protein groups and majority protein groups (MPGs) comprising all proteins sharing at least 50% of their peptides (31). This additional filter increases confidence in the identifications, and only MPGs were considered in subsequent statistical analyses. Proteins identified as contaminants and false positives were removed. Hereafter, where possible we use the term protein in exchange of MPG, as it provides a more intuitive biological description. Protein abundance values for each sample were generated using the label-free quantification (LFQ) method (32).

### Statistical Analyses

Among 604 unique proteins initially identified by MaxQuant (**Table S1**), we retained those with abundance (LFQ) values in at least seven out of ten samples across the post-immunization timecourse for all six individuals (278 MPGs; **Table S2**). Our rationale for this cut-off was to filter the analysis to only consistently measured proteins, allowing reliable downstream inferences. After filtering, just 130 missing values remained in the dataset (0.78% of 16,680 data-points) across individuals. LFQ values for the remaining proteins were log_2_-transformed and missing values imputed using a random forest-based method (33). Subsequent statistical analyses were performed on transformed imputed values using Minitab v.19 and R v3.6.2.

Principal component analyses (PCA) were performed in R using the *prcomp* function and visualized in ggplot2 v3.2.1 (34). One-way analysis of variance (ANOVA) was performed in Minitab on abundance levels for each of the 278 proteins separately, using sampling day as a fixed factor. The Anderson-Darling test was performed to verify that the residuals were normally distributed and Levene’s test used to assess homogeneity of variance. We recorded the R^2^ value for each analysis, defining the proportion of variance in protein abundance explained by sampling day. We classified proteins of interest as markers for population responses to immunization as those showing a Benjamini-Hochberg corrected P-value ≤0.05 (corrected at the level of 278 separate analyses), indicating an effect of sampling day. The relationship between P-value and R^2^ is shown in **Figure S1**, where a Benjamini-Hochberg corrected P-value ≤0.05 corresponds to R^2^ >0.34. The ANOVA data are provided in **Table S3**. For 41 proteins meeting the cut-off, differences in abundance across days were assessed using Tukey’s test. Hierarchical clustering analysis was performed in PermutMatrix (35) using abundance data for the 41 proteins, in addition to the mean total IgM abundance (mean log_2_ transformed imputed values for all fish, at each time point, for proteins in the dataset annotated as IgM) and antigen-specific Ig titres assessed by ELISA. Clustering and seriation were based on Pearson’s correlation coefficient dissimilarity z-scores. The multiple-fragment heuristic seriation method was used with complete linkage (furthest neighbour) aggregation to obtain hierarchical clusters.

### Annotation of Trout Plasma Proteins

We used STRING (36) to annotate the 278 proteins in the filtered rainbow trout plasma proteome set on the basis of identifiable orthologues in human, testing for statistical enrichment in protein-protein interaction (PPI) networks, done using default parameters, and recording enrichment (*P<*0.0001) for Biological Processes within the Gene Ontology (37) and Reactome Pathway (38) frameworks (**Table S4**). We also used the GO FEAT (39) webserver to summarize the annotation of GO Biological Processes for the same protein set.

### Analysis of Duplicated Proteins

The common ancestor to salmonid fishes underwent a whole genome duplication (WGD) event (Ss4R) ~88–103 million years ago (40) and consequently, approximately half of the functional genes are found in duplicated pairs (41, 42). Duplicated proteins retained from Ss4R share identities ranging from ~75% to >99% (41). Previous LC-MS studies in salmonids have not tested how analysis platforms using the protein group approach handle the presence of duplicated proteins. We thus aimed to establish how MaxQuant organizes duplicated proteins into MPGs using the current dataset on the basis of protein similarity (**Table 1**; **Figure S2; Tables S5, S6**).

**Table 1:** Comparison of amino acid sequence identity and shared peptide content between Ss4R and other duplicates classified into the same or different MPGs.

	% Similarity*Mean*	% Similarity*S.D.*	% Similarity*Range*	% Shared peptides*Mean*	%Shared peptides*S.D.*	% Shared peptides*Range*
*Duplicates in same MPG* (Scenario i)						
Ss4R duplicates	95.28	4.65	80.34 - 100	92.91	11.36	46.47 - 100
Other duplicates	94.66	7.17	73.21 - 100	91.12	12.93	53.13 - 100
*Duplicates in different MPGs* (Scenario ii)						
Ss4R duplicates	90.40	6.10	70.20 - 98.58	34.49	28.76	0 - 100
Other duplicates	92.16	5.89	77.34 - 98.86	43.65	33.43	0 - 100

The analysis was performed using the unfiltered high-confidence 604 MPGs and compared the number of MPGs containing duplicated proteins (“scenario i”) with the number of duplicated proteins classified into unique MPGs (“scenario ii”). The analysis was informed by BLASTp searches (43) using one representative protein per MPG as the query versus all other proteins within the full set of MPGs. For each MPG, we checked if the proteins present were translated from distinct NCBI RefSeq genes (i.e. duplicated genes), as opposed to representing isoforms of the same gene. For MPGs solely comprised of proteins translated from the same gene, BLASTp was used to record any duplicated proteins within other MPGs (cut-off: >70% identity/coverage). We used ClustalW sequence alignment (44) within BioEdit (v.7.0.5) (45) to align putative duplicated proteins within MPGs and between MPGs. The alignments were trimmed to the shortest protein present, and the mean pairwise identity between duplicated proteins was recorded. Chromosomal locations of genes encoding duplicated proteins were recorded to record duplications resulting from Ss4R (after (29, 42)). Finally, the number of detected peptides shared by duplicated proteins was recorded.

### Phylogenetic Analysis

Phylogenetic analyses were used to clarify the evolution of several proteins detected in the trout plasma proteome. For the analysis of apolipoprotein proteins we used human A-I, A-IV, and E protein sequences (NCBI accession numbers: AAA35545, AAA96731, and NP_000032, respectively) as BLASTp queries (43) against the NCBI nr protein database and collected the highest scoring homologues (e-value <1e^-10^) from coelacanth (*Latimeria chalumnae*), spotted gar (*Lepisosteus oculatus*), zebrafish (*Danio rerio*), northern pike (*Esox lucius*), Atlantic salmon (*Salmo salar*) and rainbow trout. We also performed phylogenetic analysis for two plasma proteins with ‘uncharacterized’ annotations (XP_021436350 and XP_021423950). For this analysis, the trout proteins were used as the query for BLASTp searches against the following taxa in NCBI: invertebrates, jawless fish, Chondrichthyes, Teleostei, Amphibia, Reptilia, Aves, and Mammalia. Hits for these ‘uncharacterized’ proteins were filtered to proteins showing >40/30% sequence coverage/identity to the query (e-value <1e^-04^). BLAST data are provided in **Table S7**.

For each analysis, sequence alignment was performed using Mafft v.7 with default parameters (46) and trees were built using the IQ-TREE maximum likelihood method (47, 48), which estimated and employed the best fitting amino acid substitution model (49). Ultra-fast bootstrapping (50) was used to generate branch support values. Consensus trees were visualised and rendered in Mega X (51). All used sequence alignments are given in **Supplementary Data 1**.

## Results and Discussion

### Antigen-Specific and Total IgM Response

ELISA demonstrated that an antigen-specific IgM response was generated by all six fish injected with HEL-CFA, however the timing and magnitude of the response varied considerably between individuals (**Figure 1**). While antigen-specific IgM levels began to increase in four of the six HEL-immunized fish between 28 and 35 days post-immunization, fish 2 and fish 3 did not show antigen-specific IgM binding appreciably above that of the CFA-only control fish even at day 42 (**Figure 1**). While all six HEL-immunized fish showed antigen-specific IgM levels significantly (3–5 fold) higher than the CFA-only control by day 84, there was an approximately 2-fold difference in ELISA signal between some of the antigen exposed individuals (**Figure 1**).

In mammals, primary antigen exposure causes a rapid increase in both total and antigen-specific IgM levels. This contrasts with sharks (i.e. cartilaginous fish), where total IgM levels remain relatively constant and it is only the monomeric antigen-specific portion that increases in concentration upon immunization (26). We were curious to establish if the HEL-specific IgM responses observed in trout were coupled to changes in total IgM, as in mammals, or were largely uncoupled, as in sharks. As the IgM proteins in our database were present as 11 distinct, closely related proteins, we took their average values at each time point to approximate the total IgM response. Antigen-specific IgM response and total IgM levels were significantly correlated for each of our 60 samples (Spearman’s Rho = 0.71, *P <*0.001; **Figure 1**).

### Overview of Trout Plasma Protein Functions

Biological processes and molecular pathways overrepresented among the 278 trout plasma proteins are shown in **Table S4**. These plasma proteins are highly biologically connected, showing 8.42 times more protein-protein interactions than expected by chance (*P <*1.0e^-16^; visualized in **Figure S3**). The overrepresented Biological Processes (GO framework) and Reactome pathways reflect the large number of proteins from, or involved in the regulation of, the complement system (e.g. 24 annotated with Reactome term ‘complement cascade’, *P* = 1.83e^-30^), diverse immune functions (e.g. 53 annotated with Reactome term ‘immune system’, *P* = 8.88e^-17^, 29 annotated with GO term ‘humoral immune response’, *P* = 4.88e^-23^, and 23 annotated with GO term ‘adaptive immune response’, *P* = 1.52^e-15^), a range of blood, blood clotting/coagulation and platelet functions (e.g. 34 annotated with Reactome term “hemostasis”, *P* = 7.78e^-19^), in addition to metabolic functions (e.g. 5 annotated with Reactome term ‘gluconeogenesis’, *P* = 8.14e^-05^). GO FEAT analysis highlighted similar terms, but also emphasised representation of proteins involved in lipid transport and metabolism, largely apolipoproteins (see later section).

### Analysis of Duplicated Proteins

In LC-MS proteomics, it is common to collapse proteins that share identical or highly similar peptides into single protein groups. This method aims to capture protein isoforms derived from the same gene, but also creates potential for proteins translated from highly similar duplicated genes to be grouped together. Logically, this situation will arise most commonly for proteins sharing high similarity, such as those translated from gene duplicates retained from Ss4R (41, 42). On these grounds, we predicted that the classification of duplicated proteins into distinct MPGs or a common MPG by MaxQuant would be a product of paralogous sequence divergence.

Among the 604 MPGs available for analysis, 46.5% were comprised of proteins without identifiable duplicates in the dataset (**Figure S2**). Among the remaining 286 MPGs, 31% included duplicated proteins translated from distinct genes (“scenario i”), whereas for the other 69%, any duplicated proteins in the dataset were in unique MPGs (“scenario ii”) (**Tables S5, S6**). Under scenarios i and ii, ~70% and ~85% of the proteins were retained from the Ss4R event, respectively (**Figure S2**). On average, duplicated proteins classified into the same MPG shared higher amino acid sequence identity and a higher percentage of shared peptides than duplicated proteins classified into two or more MPGs (**Table 1**). Under both scenarios we observed a significant positive correlation between the proportion of shared peptides and % identity between duplicated proteins (**Figure S4**; Pearson’s R=0.62 and 0.70; *P*=0.006 and *P*<0.0001 for scenario i and ii, respectively). MaxQuant classified many duplicated proteins sharing >95% amino acid level into distinct MPGs, despite sharing >50% common peptides (**Figure S4**). Overall, these data indicate that MaxQuant often distinguishes protein duplicates across a range of sequence divergence levels relevant to the detection of Ss4R duplicates (40, 41). However, the results advocate for a careful approach when drawing conclusions about duplicated proteins using the protein group concept in LC-MS proteomics, requiring a protein-by-protein investigation of the make-up of protein groups.

### Individual-Level Variation Dominates the Plasma Proteome

**Figure 2:**
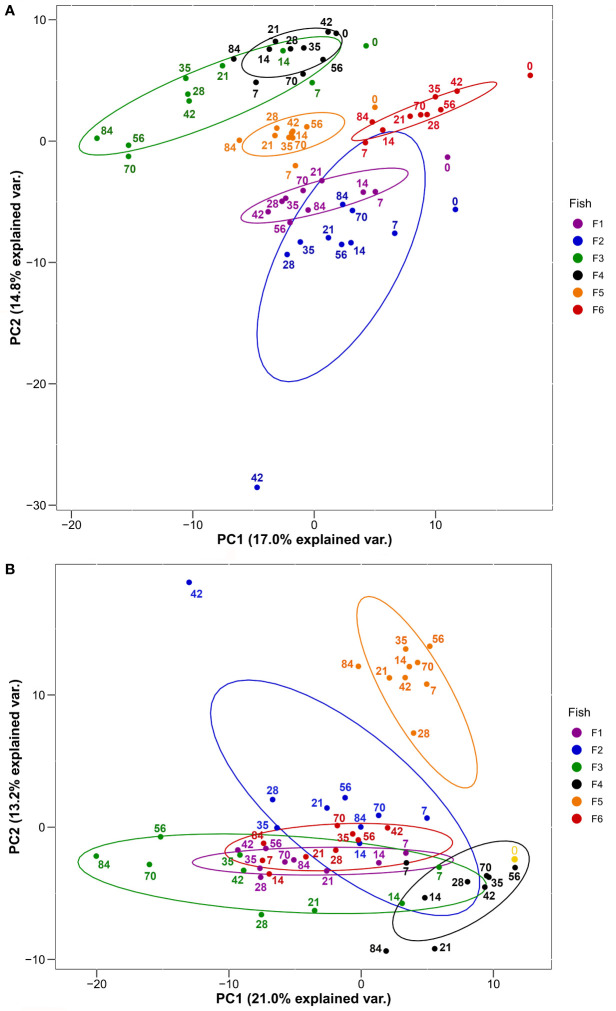
PCA of 278 plasma proteins derived from the six HEL-immunized fish shown in **Figure 1** using **(A)** log_2_-transformed imputed values and **(B)** log_2_-transformed imputed values normalized to Day 0 values. Normalized Day 0 value common to all fish is shown in yellow. Ellipses are 95% confidence intervals around the centroid. Sampling days are shown by numbering within the plots.

We used PCA to visualise groupings among the 60 sampled plasma proteomes according to fish individual and the ten post-immunization time points (**Figure 2**). We did this twice, firstly with the overall plasma protein abundance levels (**Figure 2A**), and secondly using the same values normalized to day 0 values (i.e. day 7/day 0, day 14/day 0, etc.) in order to remove the effect of variability in starting abundances, thus focusing on the response dynamic (**Figure 2B**). For the first analysis, PC1 and PC2 together explained around a third of the total variation and showed largely fish-specific groupings (**Figure 2A**). For the second analysis, a similar amount of total variation was explained by PC1 and PC2, and while there was a more substantial overlap among fish individuals (**Figure 2B**), this was not clearly explained by differences among days. Overall, a substantial proportion of total variation in our dataset is explained by overarching differences in plasma protein abundance and responses among the six trout individuals.

### Proteins Showing Similar Population Responses to Immunization

**Figure 3:**
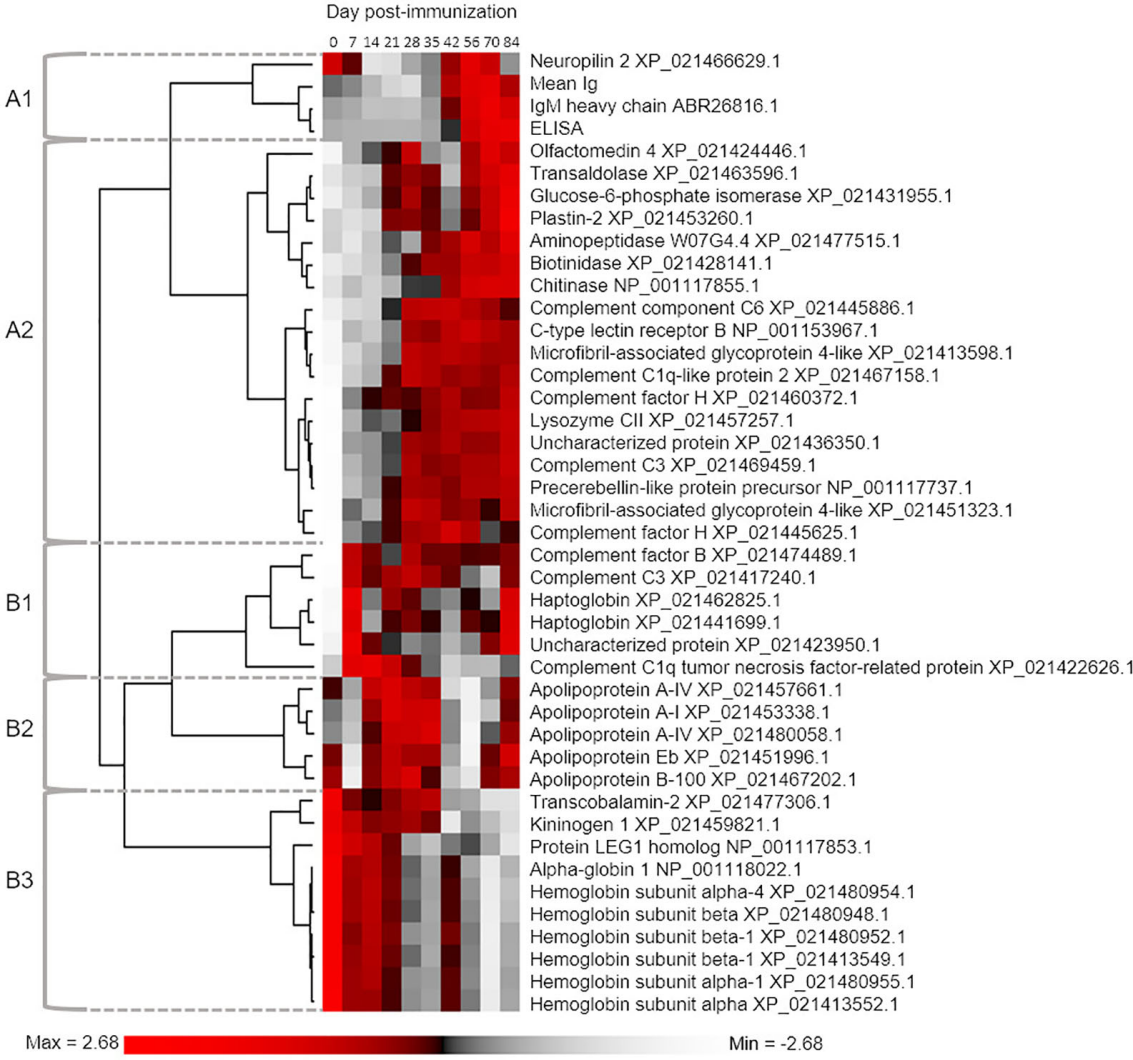
Hierarchical clustering analysis used to group proteins according to similar mean abundance profiles across the sampling timecourse. The 41 included proteins were defined by One-way ANOVA defining an overall population effect of sampling day (see **Table S3**). For comparison, we also included data for mean total Ig levels and antigen-specific IgM levels. The five defined clusters are referred to in the main text.

We next classified proteins according to the extent of variation in abundance levels explained by sampling day using one-way ANOVA (**Table S3**). We recorded R^2^ to describe the variance explained by differences among sampling days. The relationship between *P*-value and R^2^ is shown in **Figure S4**, which highlights the cut-off we employed (corrected *P ≤* 0.05) to identify 41 proteins showing an overall population effect of sampling day. These proteins represent approximately 15% of all detected plasma proteins, and within our dataset are characterized by the most similar overall abundances within days, and most similar responses to immunization across the six individuals between days. We grouped these proteins according to commonalities in their average response to immunization using hierarchical clustering, also including mean total Ig and antigen-specific IgM levels (**Figure 3**). This revealed two major clades (“A” and “B”), separating proteins showing highest abundances during the early or late part of the timecourse (**Figure 3**). In the following sections, we outline the responses and characteristics of proteins grouped into five sub-clusters, two within clade A (clusters A1 and A2) and three within clade B (clusters B1, B2, and B3).

### Cluster A1: IgM and Neuropilin-2

**Figure 4:**
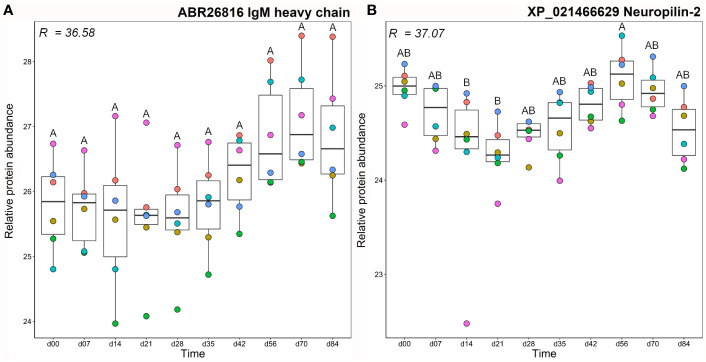
Abundance profiles for all proteins in cluster A1 (**Figure 3**); **(A)** IgM heavy chain and **(B)** neuropilin-2. R^2^ values describe the proportion of variance in abundance explained by differences in sampling day according to one-way ANOVA (**Table S3**). Different letters shown on the plots indicate days with significantly different protein abundance values (Tukey’s test). Relative protein abundance represents log2 transformed imputed values. The different colour dots represent different fish.

Mean total IgM and antigen-specific IgM clustered closely, together with a single IgM heavy chain protein (**Figure 3**), which showed large variation between fish individuals, such that abundance levels were not significantly different between any time points (**Figure 4A**). The other protein in this cluster, neuropilin-2, showed a significantly higher abundance at day 56 compared to days 14–21 (**Figure 4B**). Neuropilin-2 is a transmembrane glycoprotein that is abundantly expressed in immune cells, and supports multiple immune functions, including antigen presentation (52, 53).

### Cluster A2: Diverse Molecules With Immune Functions; From Complement to Metabolic Enzymes

**Figure 5:**
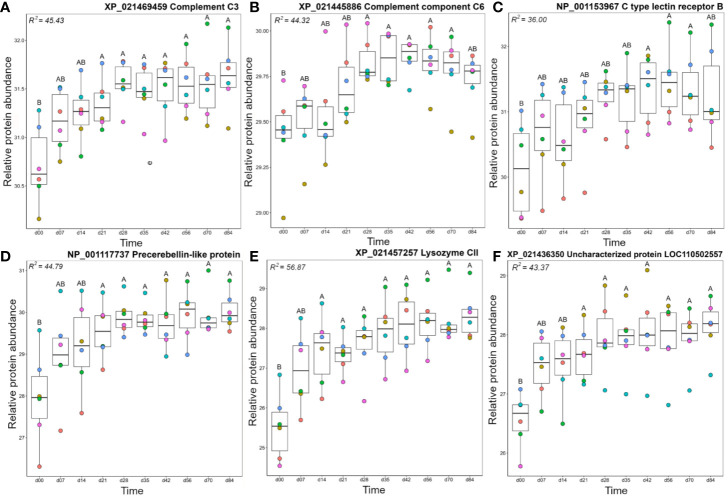
Abundance profiles for representative proteins in cluster A2 (**Figure 3**); **(A)** complement C3, **(B)** complement C6, **(C)** C type lectin receptor B, **(D)** precerebellin-like protein, **(E)** Lysozyme CII, and **(F)** uncharacterized protein XP_021436350. Other details as in the **Figure 4** legend. Additional proteins represented in cluster A2 are plotted in **Figure S5**.

Members of the A2 cluster showed lowest abundance during the earliest days in the timecourse (**Figure 3**) and included complement system proteins annotated as C1q-like, C3, C6, and factor H (**Figure 5**). For instance, a C3 protein (XP_021469459.1), which is a central component of the complement system, showed significantly higher levels on days 21–84 than day 0 (**Figure 5A**). Similarly, complement C6, part of the membrane attack complex (54), was significantly higher on days 28–70 than day 0 (**Figure 5B**). Similar abundance profile changes were observed for complement C1q-like protein 2 and two distinct factor H proteins (**Figure S5**). Several additional proteins with known immune functions were present in cluster A2, including C type lectin receptor B (**Figure 5C**), a pattern recognition receptor, precerebellin-like protein, a known acute phase protein showing regional similarity to C1q (**Figure 5D**) (55, 56) and the antibacterial enzyme lysozyme (57) (**Figure 5E**). Two separate microfibril-associated glycoprotein 4 proteins also grouped within cluster A2 (**Figure S5**), and may act as soluble pathogen recognition molecules (58). Cluster A2 also included a protein annotated ‘uncharacterized’ (XP_0214236350; detailed further below) that showed significantly higher abundance at days 21-84 compared to day 0 (**Figure 5**).

**Figure 6:**
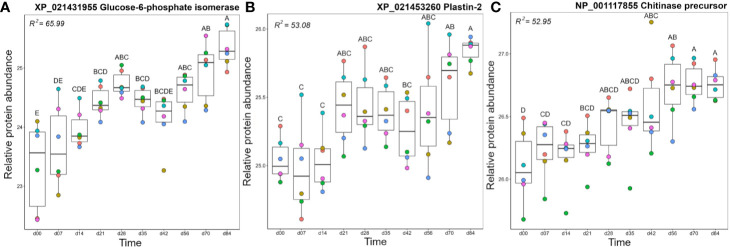
Abundance profiles for representative proteins in cluster A2 (**Figure 3**); **(A)** Glucose-6-phosphate isomerase, **(B)** Plastin-2, and **(C)** Chitinase precursor. Other details as in the **Figure 4** legend. Additional proteins represented in cluster A2 are plotted in **Figure S5**.

Within cluster A2, glucose-6-phosphate isomerase (GPI) levels increased steadily during the timecourse, and reached peak levels at day 84, which was significantly higher than days 0-42 (**Figure 6A**). While best known for its role in glycolysis and gluconeogenesis, in mammals a secreted form of GPI (“neuroleukin”) induces maturation of B cells into plasma cells, thereby increasing Ig production (59). Thus, the increasing levels of GPI may have been supporting the development of the observed antigen-specific IgM response. Like GPI, plastin-2 (LCP1 or L-plastin) increased during the timecourse to show highest levels at day 84, with days 70 and 84 each showing significantly higher levels than days 0–14 (**Figure 6B**). Plastin-2 is an actin-regulating protein expressed specifically in leucocytes, and in humans is among the most abundant of all monocyte and T-cell proteins, with key immune functions including lymphocyte migration and T-cell activation (60, 61). The metabolic enzyme transaldolase showed a similar abundance profile (**Figure S5**), and is involved in the pentose phosphate pathway; its increasing levels during the time course may help support the high metabolic demands of an adaptive immune response (62).

Olfactomedin-4 showed a significant increase in abundance at days 28 and days 56–84 compared to day 0 (**Figure S5**). The secreted form of olfactomedin-4 can interact with many proteins, such as NOD1/NOD2, lectins, cadherins, and cathepsins, all of which regulate immune functions (63). The enzymes chitinase (**Figure 6C**), biotinidase (**Figure S5**), and aminopeptidase W07G4.4 (**Figure S5**), all increased steadily in abundance across the timecourse, with chitinase and biotinidase reaching significantly higher levels than day 0 by days 35–42. Chitinase has well established roles in innate immune protection, providing antifungal and antihelminthic activity (64). While no direct immune functions have been attributed to biotinidase, this enzyme may support immune function indirectly, e.g. biotin is an essential cofactor for enzyme functioning in many cell types, including maturing and proliferating lymphocytes (65, 66). Upregulation of biotinidase should increase the supply of biotin, perhaps supporting the production of antigen-specific Ig in the adaptive phase.

### Cluster B1: Including Haptoglobins and Complement Proteins

**Figure 7:**
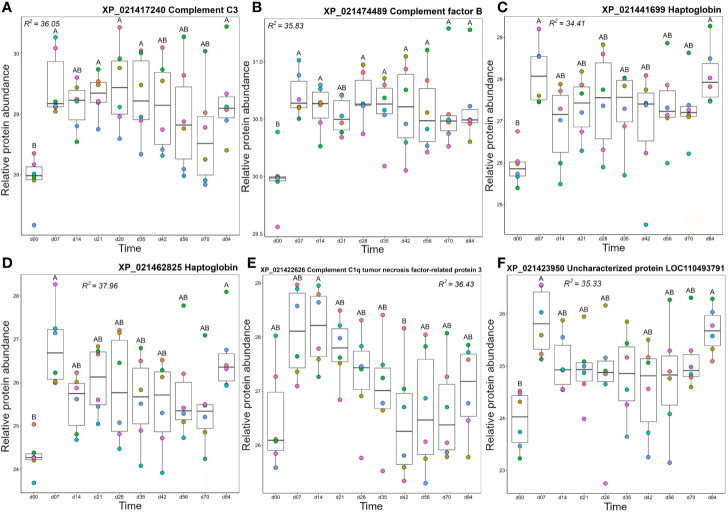
Abundance profiles for all proteins in Cluster B1 (**Figure 3**); **(A)** complement C3, **(B)** complement factor B, **(C)** haptoglobin, **(D)** haptoglobin, **(E)** complement C1q tumor necrosis factor-related protein 3, and **(F)** uncharacterized protein XP_021423950. Other details as in the **Figure 4** legend.

Cluster B1 contained proteins that tended to show highest levels from day 7 onwards (**Figure 3**). This included two complement proteins, a second C3 molecule (XP_021417240) along with factor B (Bf), an integral component of the alternative pathway C3 convertase (67). Both molecules showed a dramatic increase in abundance between day 0 and 7 and remained at elevated levels across the remainder of the timecourse (**Figures 7A, B**). Cluster B1 contained two haptoglobin (Hp) molecules, also showing large increases in abundance from day 0 to 7, consistent with Hp being an acute-phase protein, before dropping back to levels not significantly different from 0 between days 14–70, and increasing again on day 84 (**Figures 7C, D**). Hp is best known for sequestering haemoglobin released by haemolysis in mammals (68). However, it has recently been shown that Hp is a divergent member of the complement-activating MASP family (69) and possesses various immunoregulatory functions (70, 71). The adipokine C1q TNF-related protein (CTPR3) showed a similar mean profile, but with larger variation among individuals, and only showed a significant difference in abundance between days 14 and 42 (**Figure 7E**). Given the wide-ranging effects ascribed to CTPR3, including roles in metabolism and inflammation (reviewed in (72)), it is difficult to predict the consequences of these changes. A second uncharacterized protein (XP_0211423950; detailed further below) was contained in cluster B1. This protein increased significantly in abundance between day 0 and day 7, dropped to a slightly lower level from days 14–70, then increased in abundance again at day 84 (**Figure 7F**), an abundance profile remarkably similar to the Hp proteins, with which it clustered (**Figure 3**).

### Cluster B2: Apolipoproteins

**Figure 8:**
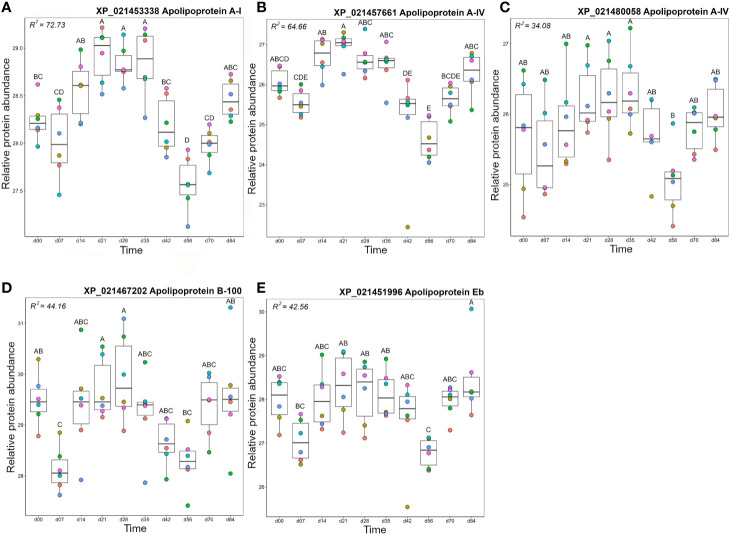
Abundance profiles for all proteins in Cluster B2 (**Figure 3**); **(A)** apolipoprotein A-I, **(B)** apolipoprotein A-IV, **(C)** apolipoprotein A-IV, **(D)** Apolipoprotein B-100, and **(E)** apolipoprotein Eb. Other details as in the **Figure 4** legend.

Cluster B2 was comprised of five apolipoproteins (**Figure 3**), among 17 unique apolipoproteins detected in our dataset (**Table S2**). Each of the five apolipoproteins showed related changes in abundance during the timecourse, but with distinct levels of individual variation within days (**Figure 8**). Two of these proteins showed among the highest R^2^ values reported in our dataset (**Table S3**; **Figures 8A, B**). Apolipoprotein A-I (XP_021453338) showed highest levels at days 21–35 (significantly higher than days 0–7 and days 42–70) and lowest levels at day 56 (significantly lower than all days except days 42 and 70) (**Figure 8A**), which was highly similar to apolipoprotein A-IV (**Figure 8B**). A distinct protein annotated as apolipoprotein A-I (XP_021480058) showed a similar profile, but with greater individual variation within days, showing a significantly lower abundance at day 56 compared to days 21–35 (**Figure 8C**). Apolipoprotein B-100 was unique in showing a significant decrease from day 0 to day 7, before significantly increasing by day 21, and significantly decreasing again by day 56 (**Figure 8D**). Apolipoprotein Eb showed significantly lower abundance at day 56 compared to days 21–35 and day 84 (**Figure 8E**).

**Figure 9:**
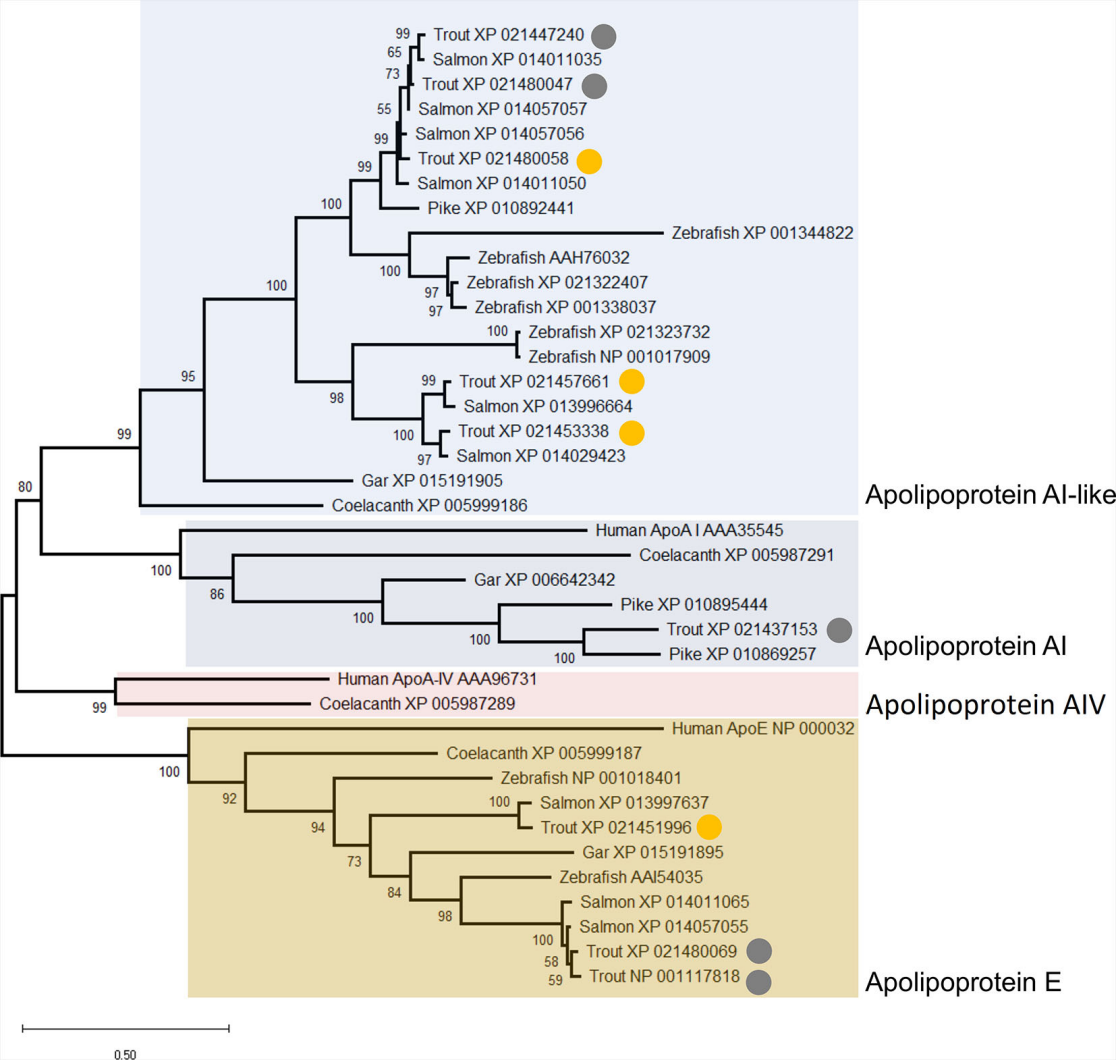
Maximum likelihood consensus tree of apolipoprotein A and E proteins detected in the rainbow trout plasma proteome, alongside putative orthologues from human, coelacanth, spotted gar, zebrafish, Atlantic salmon, and northern pike. The tree, which is rooted to the ApoE clade, was generated using the best fitting amino acid substitution model (LG+G4) and includes branch support values from 1,000 ultrafast bootstrap replicates. The circles highlight nine distinct apolipoprotein A and E family members detected in our dataset. The yellow circles highlight proteins included in Cluster B2 (**Figure 3**) with specific abundance profiles plotted in **Figure 8**.

The similar annotations and abundance profiles of multiple apolipoprotein A family members led us to question their evolutionary relationships. A phylogenetic analysis was performed for all annotated apolipoprotein A proteins in our dataset, within a broader background of orthologs from additional vertebrate species (**Figure 9**). Note, we included the apolipoprotein E family in this analysis as the sister group to the apolipoprotein A family, but excluded apolipoprotein B molecules, which are more evolutionarily divergent (73). This analysis provides a different, and more well supported evolutionary history of apolipoprotein AI and IV than existing scenarios (73). Specifically, our tree strongly supports a scenario where the ancestor to ray-finned fish and lobe-finned fish had separate genes encoding three distinct proteins related to apolipoprotein AI and IV, all of which are retained in coelacanth (**Figure 9**). Included among these three proteins, humans retain apolipoprotein AI and IV while ray-finned fish only retain orthologues of AI (**Figure 9**). However, ray-finned fish and coelacanth retain a distinct paralogue of apolipoprotein AI, absent in humans (**Figure 9**). Three rainbow trout representatives of this clade are grouped within Cluster B2, with two representing salmonid-specific duplicates (i.e. XP_021453338 and XP_021457661) (**Figure 9**) showing highly correlated abundance changes (**Figure 3**). The third protein (XP_021480058) is a more ancient paralogue that arose in the common ancestor to salmonids and zebrafish after the split from spotted gar, which is consistent with the teleost-specific WGD event (74). The current annotation of apolipoprotein A-like proteins in salmonids is thus not supported by phylogenetic analysis, and future work might expand the phylogenetic analysis of the apolipoprotein A family to achieve an evolutionarily-supported nomenclature.

When interpreting the changes in abundance of apolipoproteins in our data, it is important to note that while classically associated with lipid transport and metabolism (75) these molecules also modulate innate and adaptive immune processes (reviewed in (76)). Recent data from amphioxus (77) suggest at least some of these immune roles are evolutionarily ancient. Apolipoprotein B-100 has been shown to inhibit *Staphylococcus aureus* colonization (78), while class A apolipoproteins can bind and neutralize bacterial LPS (79) and have bactericidal (80–82) and viricidal (83) activity in teleosts, including salmonids. They also kill pathogens indirectly, acting as immunostimulants to enhance respiratory burst responses in innate immune cells (84). Class A and E apolipoproteins also modulate many adaptive immune processes. Generally these immune functions tend towards regulation, e.g. inhibiting the production of pro-inflammatory cytokines by disrupting contact between stimulated T cells and monocytes (85), stimulating regulatory T cell expansion (86), and inhibiting T cell activation through the downregulation of antigen-presentation by dendritic cells (87). Thus, the complex changes in abundance observed across the timecourse may reflect the multifaceted regulatory roles of rainbow trout apolipoproteins at different phases of the immune response.

### Cluster B3: Including Haemoglobin and Proteins With Blood Functions

**Figure 10:**
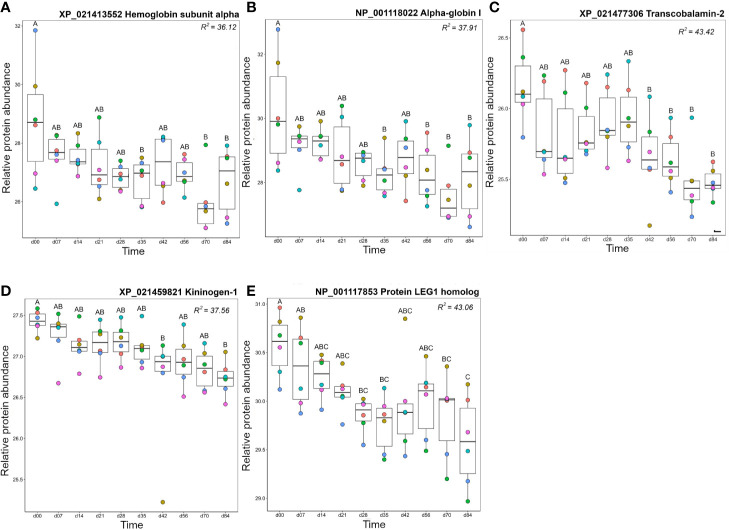
Proteins in Cluster B3 (**Figure 3**); **(A)** hemoglobin subunit alpha, **(B)** alpha-globin I, **(C)** transcobalamin-2, **(D)** kininogen-1, and **(E)** protein LEG1 homolog. Other details are as in the **Figure 4** legend.

Proteins in cluster B3 showed highest levels on day 0, then decreased in abundance over the timecourse (**Figure 10**, **Figure S6**). Seven haemoglobin (Hb) proteins fell within this cluster and reached lowest levels on day 70. The presence of Hb in plasma is due to red blood cell (RBC) lysis, occurring biologically *in vivo* or during sample processing *ex vivo*. The drop in abundance of Hb subunits may simply reflect improvement in our sampling and processing technique over the experiment, however, there are several biological processes that could cause or contribute to this drop. For example, repeated blood sampling could reduce RBC counts and consequently reduce free Hb. However, Collet and colleagues (12) recorded no significant drop in haematocrit using the same approach with more regular bleeding (taken every 4 days). While we did not measure haematocrit, the fish in our study were sampled every 7 days using the same volume criteria (<10% blood volume from each animal in any 4-week period). Thus, it would be surprising if reduced Hb was caused by reduced haematocrit. Replacement of removed RBCs with newer, more robust, cells could partly explain the drop in free Hb. Finally, there is known to be a slow turn-over of RBCs *in vivo* due to formation of the alternative pathway C3 convertase (formed from fragments of C3 and Bf) of the complement system on their surface. It is worth noting that free Hb levels drop around the time that the levels of one C3 and Bf decrease, while the two molecules of factor H, a regulatory protein which accelerates the decay of the C3 convertase and thus would protect host RBCs from lysis, show an increase. Further study is required to ascertain which, if any, of these mechanisms explain our data.

Transcobalamin-2, which is required to bind and transport vitamin B12 for red blood cell production (88, 89), also decreased over the timecourse, with highest abundance levels between day 0 and day 35, dropping at day 42 and remaining at this level until day 84 (**Figure 10C**). Kininogen-1 is a multi-functional protein with a major role in blood clotting and blood pressure regulation but is also a mediator of immune inflammation (90). Kininogen-1 levels decreased steadily over the experimental timecourse such that there was a significant drop between day 0 and day 84 (**Figure 10D**). Protein LEG1 homolog, a structural protein necessary for liver development and function (91), also decreased in abundance over the study timecourse, showing lower levels at days 70–84 than at day 0 (**Figure 10E**).

### Evolutionary Conservation of “Uncharacterized” Trout Plasma Proteins

**Figure 11:**
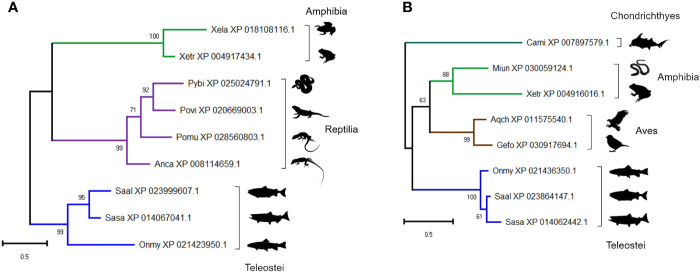
Maximum likelihood consensus trees for two trout proteins annotated as ‘uncharacterized’: **(A)** XP_021436350 and **(B)** XP_021423950, including putative orthologues in other taxa. The trees were generated using the best fitting amino acid substitution model and includes branch support values from 1,000 ultrafast bootstrap replicates. Species abbreviations: Anca, Anolis carolinensis (green anole); Aqch, Aquila chrysaetos (Golden eagle); Cami, Callorhinchus milii (elephant shark); Gefo, Geospiza fortis (medium ground finch); Miun, Microcaecilia unicolor (caecilian spp.); Onmy, Oncorhynchus mykiss (rainbow trout); Pomu, podarcis muralis (common wall lizard); Povi, Pogona vitticeps (bearded dragon); Pybi, Python bivittatus (Burmese python); Saal, Salvelinus alpinus (Arctic charr); Sasa, Salmo salar (Atlantic salmon); Xela, Xenopus laevis (African clawed frog); Xetr, Xenopus tropicalis (Western clawed frog).

The presence of two proteins annotated as ‘uncharacterized’ within the group of 41 proteins included in **Figure 3** (**Figures 5F**, **8F**), led us to question their annotations and evolutionary origins. We determined if these molecules show homology to proteins in other taxa using BLASTp (**Table S7**). Despite not being detected in any mammal, both proteins are conserved across multiple vertebrate classes (**Table S7**). The protein in cluster A2 (XP_021436350) is a 22 kDa cysteine-rich glycoprotein containing no known conserved domains, conserved in teleosts, amphibians and reptiles (**Figure 11A**). The protein in cluster B1 (XP_021423950) is a 24 kDa cysteine-rich protein with no known conserved domains, and conserved in cartilaginous fish, birds, and teleosts (**Figure 11B**). The identification of conserved uncharacterized proteins showing temporal changes in abundance highly correlated to known immune molecules highlights a strength of the “discovery” approach enabled by LC-MS proteomics; specifically, the scope for revealing novel molecules with putative immune functions, which clearly warrant further attention.

## Conclusions, Perspectives, and Caveats

This is the first study to apply label-free proteomics to quantify long-term changes in plasma protein abundance in a salmonid fish following immunization, capturing different phases in the immune response. By keeping samples from each individual separate, we were able to explore the variation in humoral response between individuals as well as population-level responses. It is well-established that vaccination does not induce comparable protective responses in individual fish (92–94), however the extent of individual variation we observed here was nevertheless surprising. For example, although all fish produced antigen-specific IgM, the kinetics and magnitude of the response varied markedly between individuals. Importantly, now we have proved antigen-specific IgM levels as measured by antigen-binding ELISA correlate highly with total IgM as measured by LC-MS/MS, future studies would require much smaller plasma volumes (i.e. only 1 μl of plasma is required for LC-MS analysis compared to ≥10 μl for ELISA) thus permitting study of immune responses in much smaller fish (e.g. fingerlings of usual vaccination size).

Mirroring the data on the IgM response, we found that the overall plasma proteome response to HEL immunization was predominantly explained by differences between fish individuals. Due to the small number of individuals in our study we did not attempt to explore the factors contributing to this variation in response. However, given the importance of consistent, population-wide protection to the eventual success of aquaculture vaccines, this is certainly something that should be explored in future studies. The power of our study comes from our ability to compare each time point from an individual animal to its own day 0 sample. We cannot rule out that some changes in protein abundance are linked to the repeated sampling protocol rather than immune challenge, the globin proteins being a possible example. However, it seems unlikely this would be the case for all the proteins showing similar abundance profiles across fish individuals, especially as many have previously established immune functions. Following additional validation, at least some of these molecules may provide useful new immune biomarkers that can be utilized in future vaccine trials in salmonids. Of note is our discovery of two uncharacterized plasma proteins with putative immune functions. Our phylogenetic analyses indicate these proteins are absent from placental mammals, including humans and mice, so likely would not have been identified using standard comparative approaches. It will be interesting to discover what role these new molecules play in immune protection in trout and, where present, other species. As yet we are unable to correlate any specific proteins with high production of antigen-specific antibody, however, as data from other proteomic studies is accrued and new markers are utilized, we are confident such information will eventually be forthcoming.

Finally, our study highlights how LC-MS is an enabling technology for the study of non-mammalian species where large sequence datasets are often available, but species-specific monoclonal antibodies are in short supply. Indeed, the power of LC-MS is illustrated by our ability to differentiate proteins with high amino acid identity, such as those expressed from recently duplicated genes. These may have different functional roles/response kinetics but would be difficult to raise discriminatory antibodies against. Even if such antibodies were available, the research effort and sample volumes required to monitor abundance changes of >250 plasma proteins renders the use of classical methods such as ELISA or Western blots impractical. However, we also recognize there is room to further improve our approach; for example, although we detected over 600 unique proteins in our samples, only 278 proteins were detected in at least seven out of ten samples for all six individuals and thus retained following filtering. This issue could perhaps be overcome through application of an isobaric labelling strategy, whereby the peptides generated following trypsin treatment of each sample are tagged with chemical groups of identical mass but differing in their distribution of stable heavy isotopes. Tagged samples are pooled then analyzed simultaneously by MS. While tagging kits can be costly, such strategies should reduce the number of missing observations and consequentially improve the number of unique proteins retained for subsequent analyses. Alternatively, another recently developed label free MS method called sequential window acquisition of all theoretical mass spectra (SWATH) can be used to increase precision across the detectable proteome [e.g. (95)]. The application of such methods would also facilitate the detection of important but low abundance proteins, such as cytokines, that were not picked up during this study. Even with such limitations, the in-depth information obtained *via* LC-MS studies such as ours will undoubtedly improve our understanding of fish immune responses and the immunological variation between individuals, hopefully accelerating the testing of new aquaculture vaccines and improved administration methods.

## Data Availability Statement

The datasets presented in this study can be found in online repositories. The names of the repository/repositories and accession number(s) can be found in the article/**Supplementary Material**.

## Ethics Statement

The animal study was reviewed and approved by UK home office and University of Aberdeen’s Animal Welfare and Ethical Review Body (AWERB).

## Author Contributions

Study conception and design: DM and HD. Animal work: MM and HD. Proteomics lab work: DS. Proteomic data analysis: FB, DC, AD. Data interpretation: FB, DM, and HD. Drafted figures and tables: FB and DM. Drafted manuscript: FB, DM, and HD. All authors contributed to the article and approved the submitted version.

## Funding

This work was supported by the Biotechnology and Biological Sciences Research Council (BBSRC) grant numbers: BB/M010996/1, BB/M026345/1, BBS/E/D/20002174, and BBS/E/D/10002071.

## Conflict of Interest

The authors declare that the research was conducted in the absence of any commercial or financial relationships that could be construed as a potential conflict of interest.
