# Supplementary material for: Plasma Proteome Responses in Salmonid Fish Following Immunization
Source: Front Immunol. 2020 Oct 8;11:581070. doi: 10.3389/fimmu.2020.581070 (PMC7579410; doi:10.3389/fimmu.2020.581070)
Supplement: Supplementary file 1 [file Table_1.docx]

Supplementary Information

**Plasma proteome responses in salmonid fish following immunization**

Bakke F.K., Monte M.M., Stead D.A., Cornulier T., Causey D.R., Douglas A., Macqueen D.J., Dooley H.

**Includes Fig. S1 – S6**

Attached in .xlsx format: **Supplementary Tables**

Table S1: **Raw proteomic data**; Label-free quantification (LFQ) values for 605 proteins identified by the MaxQuant Andromeda peptide search engine. Contaminants and reverse sequence counterparts are not shown.

Table S2: **Filtered proteomic data**; All proteins with LFQ values for at least 7 out of 10 time points across the post-immunization time course.

Table S3: **Statistical analysis of filtered dataset**; using one-way ANOVA for the filtered dataset of 278 proteins

Table S4: **STRING enrichment analysis**; for the filtered dataset of 278 proteins

Table S5: **Analysis of duplicated proteins, scenario i** ; Duplicated proteins present in the same majority protein group

Table S6: **Analysis of duplicated proteins scenario ii**; Duplicated proteins present in unique majority protein groups

Table S7: **Uncharacterized protein analysis**; Results of BLAST queries against two proteins in the filtered dataset which were annotated as uncharacterized in rainbow trout

Attached in .txt format: **Supplementary Data**

Supplementary Data 1: **Sequencing alignments used in study**


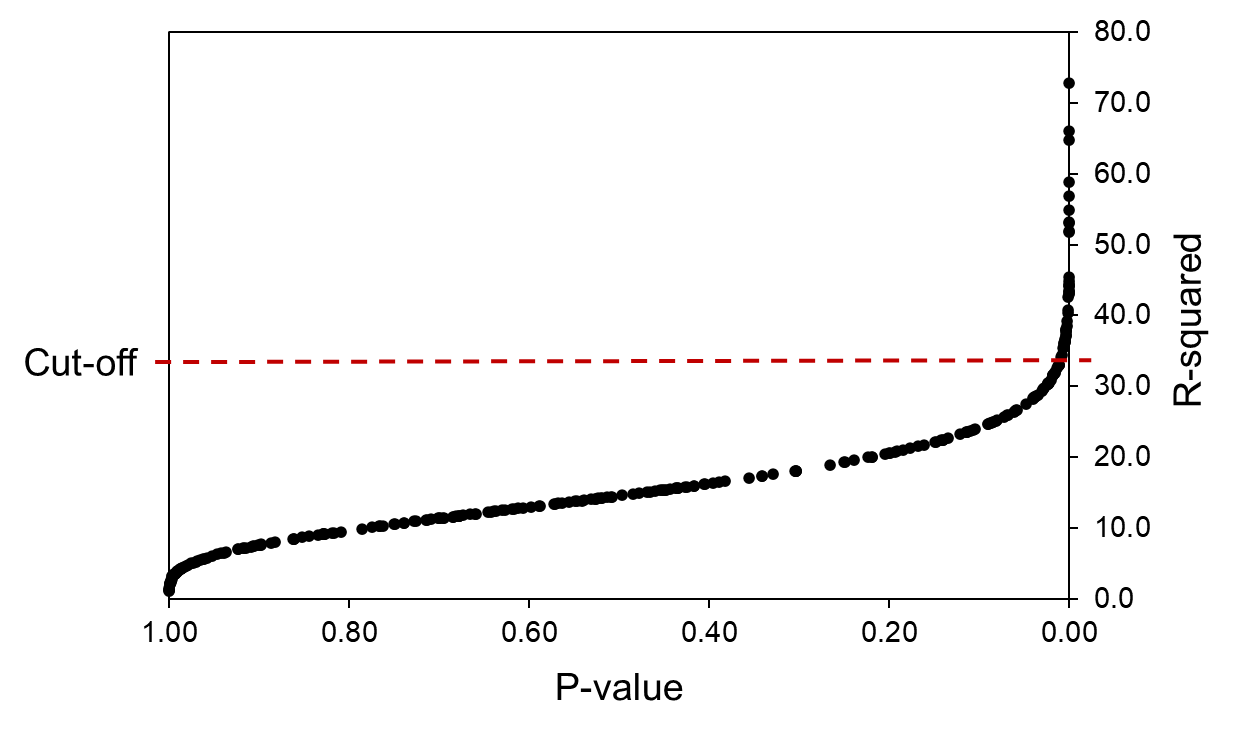


**Fig. S1**. Scatterplot showing relationship between R^2^ and P-value following one-way ANOVA (time as fixed factor) of 278 plasma proteins, highlighting the cut-off used (*P<*0.05 after Bonferroni-Holm correction based on 278 separate analyses) for further investigation of proteins.


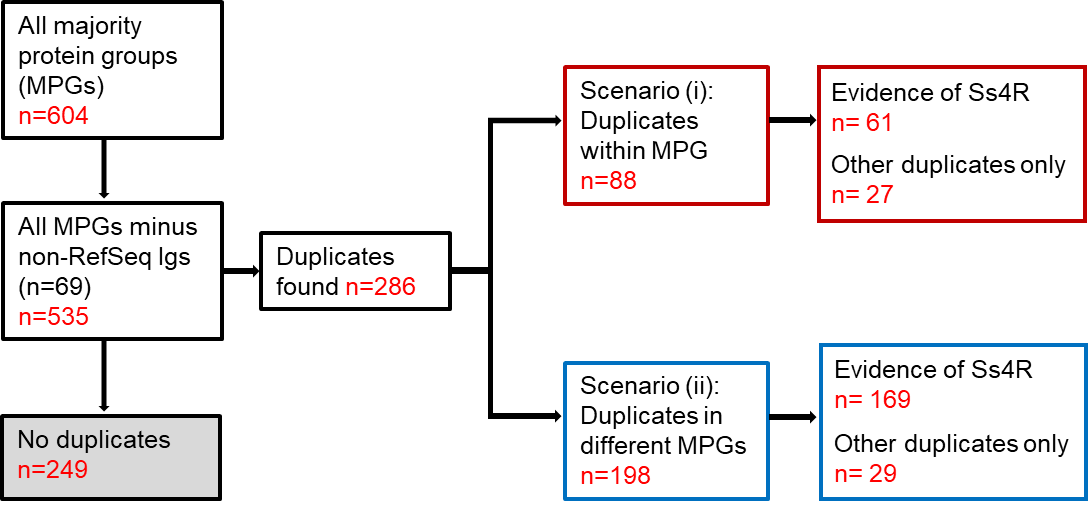


**Fig. S2**. Visualization of approach used to determine how MaxQuant groups duplicated proteins into common or different protein groups.


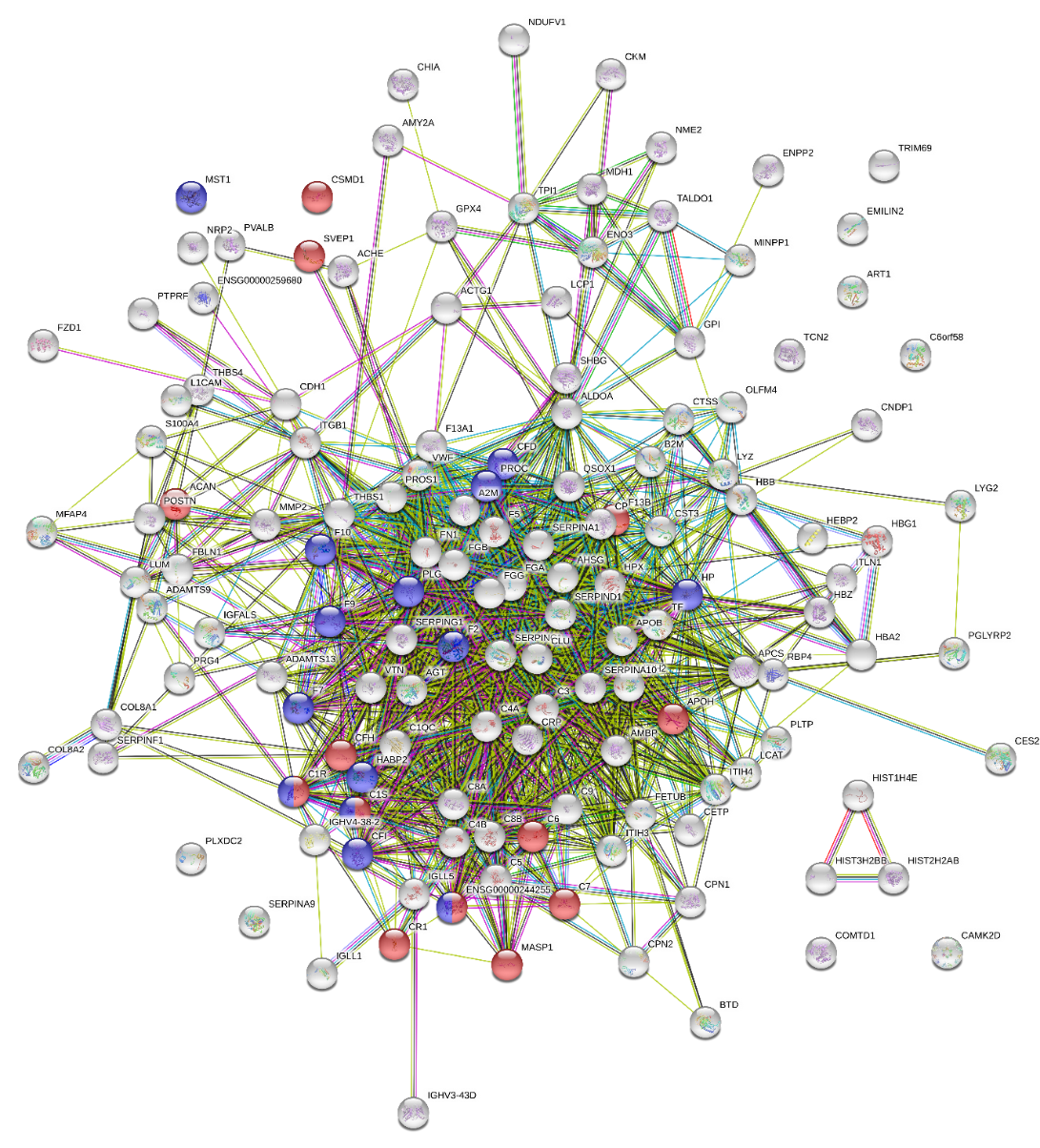


**Fig. S3**. STRING protein-protein interaction (PPI) network for putative human orthologues of the filtered trout plasma protein dataset. PPI enrichment = *P* < 1.0e-16.


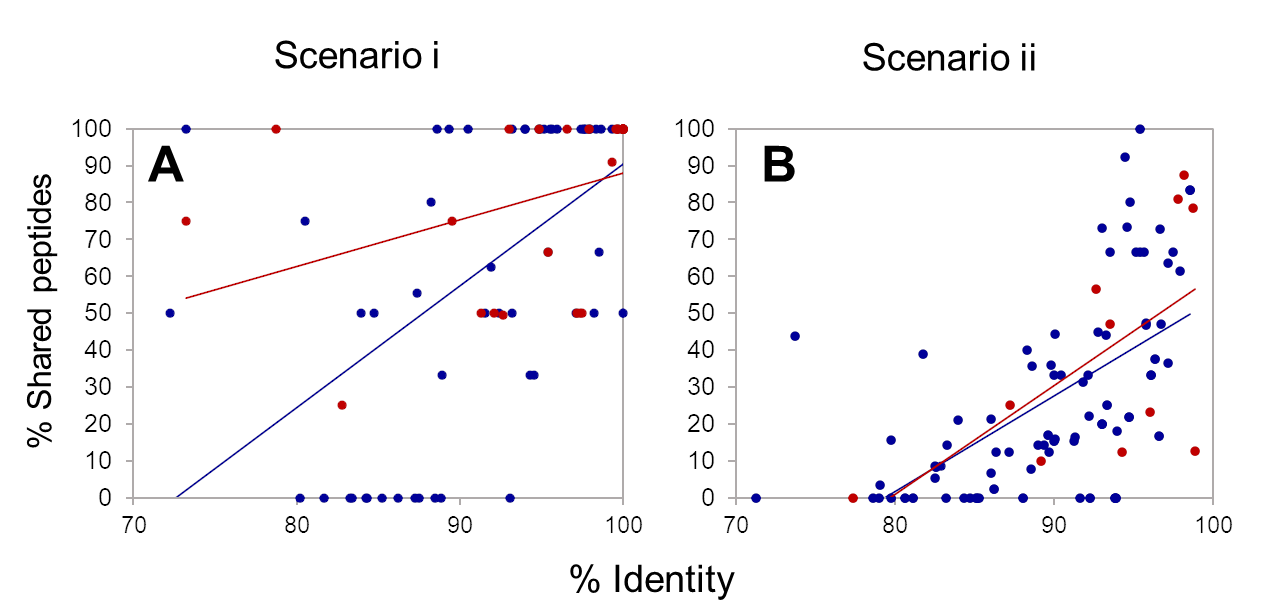


**Fig. S4**. Relationship between protein sequence identity and proportion of shared peptides among duplicated proteins classified into (**A**) scenario i, the same MPG and (**B**) scenario ii, unique MPGs. Colours show duplication category (red = Ss4R; blue = other duplications).

**
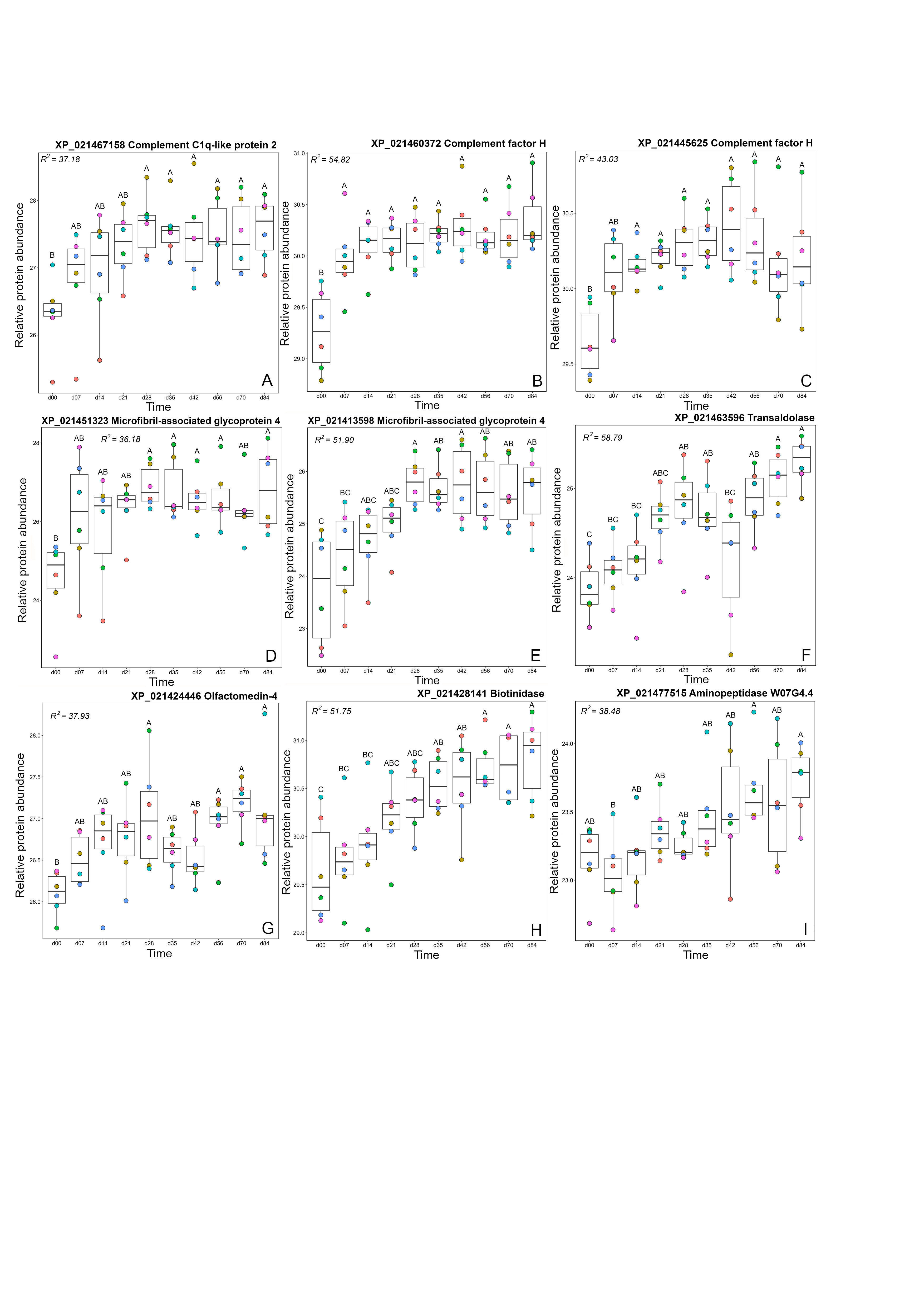
**

**Fig S5.** *Additional proteins represented in cluster A2 (see Fig. 5, main text)*


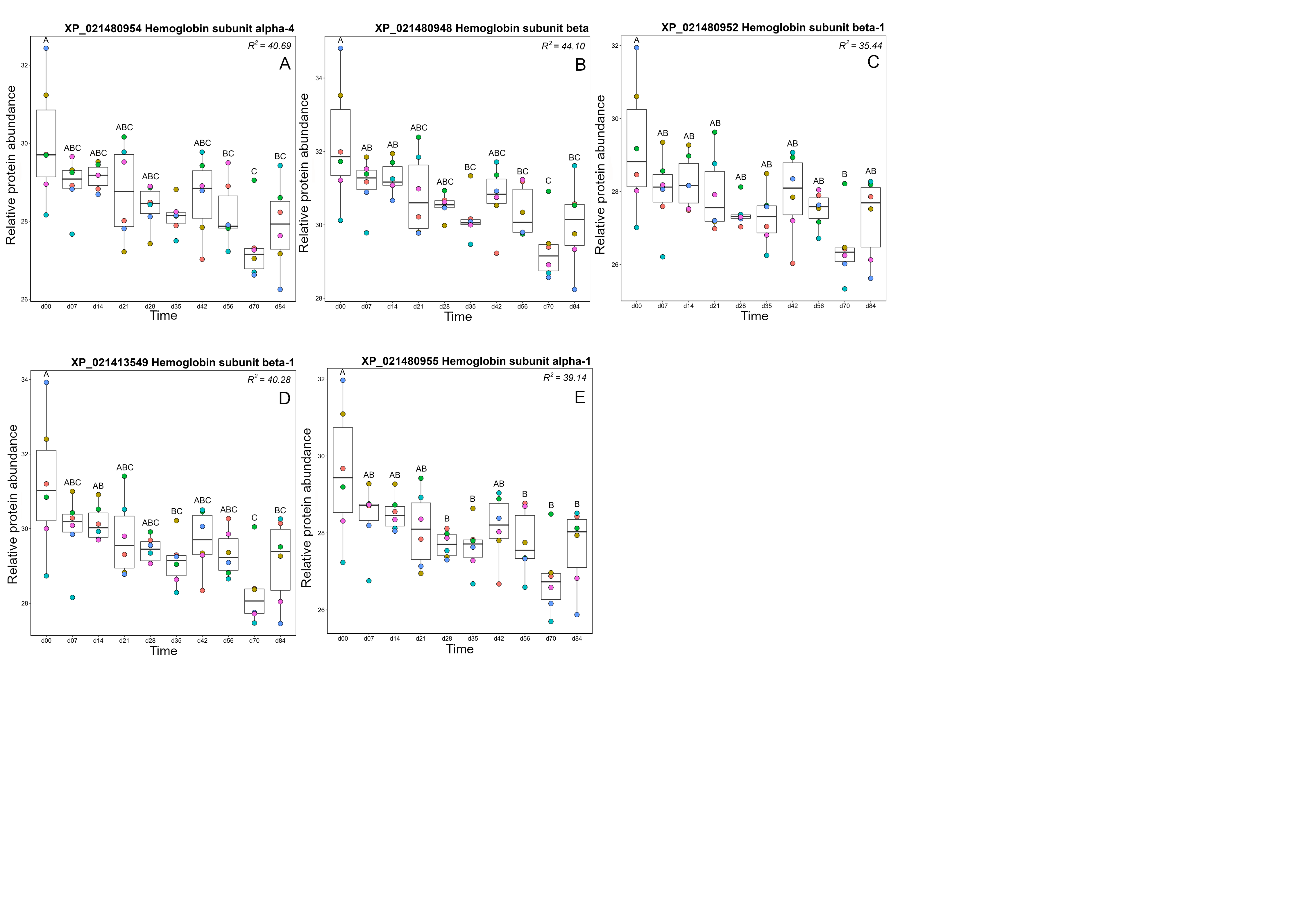


**Fig S6.** *Additional proteins represented in cluster B3 (see Fig. 10, main text)*
